# APOC1 as a novel diagnostic biomarker for DN based on machine learning algorithms and experiment

**DOI:** 10.3389/fendo.2023.1102634

**Published:** 2023-02-20

**Authors:** Kuipeng Yu, Shan Li, Chunjie Wang, Yimeng Zhang, Luyao Li, Xin Fan, Lin Fang, Haiyun Li, Huimin Yang, Jintang Sun, Xiangdong Yang

**Affiliations:** ^1^ Department of Nephrology, Qilu Hospital of Shandong University, Jinan, Shandong, China; ^2^ Department of Blood Purification, Qilu Hospital of Shandong University, Jinan, Shandong, China; ^3^ Laboratory of Basic Medical Sciences, Qilu Hospital of Shandong University, Jinan, Shandong, China; ^4^ Department of Geriatric Medicine, Qilu Hospital of Shandong University, Jinan, Shandong, China; ^5^ Department of General Practice, Qilu Hospital of Shandong University, Jinan, Shandong, China

**Keywords:** DN, biomarker, diagnostic, machine learning algorithms, APOC1

## Abstract

**Introduction:**

Diabetic nephropathy is the leading cause of end-stage renal disease, which imposes a huge economic burden on individuals and society, but effective and reliable diagnostic markers are still not available.

**Methods:**

Differentially expressed genes (DEGs) were characterized and functional enrichment analysis was performed in DN patients. Meanwhile, a weighted gene co-expression network (WGCNA) was also constructed. For further, algorithms Lasso and SVM-RFE were applied to screening the DN core secreted genes. Lastly, WB, IHC, IF, and Elias experiments were applied to demonstrate the hub gene expression in DN, and the research results were confirmed in mouse models and clinical specimens.

**Results:**

17 hub secretion genes were identified in this research by analyzing the DEGs, the important module genes in WGCNA, and the secretion genes. 6 hub secretory genes (APOC1, CCL21, INHBA, RNASE6, TGFBI, VEGFC) were obtained by Lasso and SVM-RFE algorithms. APOC1 was discovered to exhibit elevated expression in renal tissue of a DN mouse model, and APOC1 is probably a core secretory gene in DN. Clinical data demonstrate that APOC1 expression is associated significantly with proteinuria and GFR in DN patients. APOC1 expression in the serum of DN patients was 1.358±0.1292μg/ml, compared to 0.3683±0.08119μg/ml in the healthy population. APOC1 was significantly elevated in the sera of DN patients and the difference was statistical significant (P > 0.001). The ROC curve of APOC1 in DN gave an AUC = 92.5%, sensitivity = 95%, and specificity = 97% (P < 0.001).

**Conclusions:**

Our research indicates that APOC1 might be a novel diagnostic biomarker for diabetic nephropathy for the first time and suggest that APOC1 may be available as a candidate intervention target for DN.

## 1 Introduction

Diabetic nephropathy (DN) is one of the most serious complications of diabetes and 45% of DN patients will progress to end-stage renal disease (ESRD) (1), which affects the quality of life and causes a substantial economic burden to society (2). The gold standard for diabetic kidney diagnosis remains renal pathology, but renal puncture biopsy methods are invasive for DN patients. In recent years, some biological signatures have been detected for the diagnosis of DN, such as KIM-1, NGAL, suPAR, YKL-40, and so on (3–5). However, there are no valid and reliable biological markers for the diagnosis of DN.

GEO Database is a database established by the National Centre or Biotechnology Information (NCBI) to determine the critical genes and underlying molecular mechanisms for disease pathogenesis and progression (6). Recently, bioinformatics and machine learning methods extensively employed in biomarker screening by using the GEO database (7–9). What’s more, secreted proteins have significance in course of biological activity, specifically in the diagnosis of diseases and future target therapies (10, 11). This provides the opportunity to detect novel plasma markers for the recognition of patients with DN.

The research aims to reveal potential predictor plasma biomarkers of DN by data mining, which will generate novel insights into the mechanisms of DN pathogenesis and provide directions for future research into alternative therapies. If the potential predictor plasma biomarkers accurately predict the probability of DN occurring, the disease may be treated with prevention and intervention at an early stage.

## 2 Materials and methods

### 2.1 DEGs data processing

Expression profiles of GSE96804 mRNA were obtained from the GEO database (GPL17586 platform, Affymetrix Human Transcriptome Array 2.0) (12). In total, 61 tissue biopsies, 41 tissue samples from DN tissue samples and 20 from the normal, were obtained from the National Clinical Research Center of Kidney Diseases, Jinling Hospital, Nanjing University School of Medicine. ^“^Limma^”^ packaged (13) in R software was used to process data and the “ggplot2” (14), “Pheatmap” packages for drawing of figures. DEGs were identified with |log Fold Change | ≥1 & adj P Val < 0.05.

### 2.2 GO and KEGG enrichment analysis

GO analysis was conducted using the ‘cluster Profiler’ (15), ‘GO plot’, and ‘ggplot2’ packages for up- and down-regulated DEGs with altered DN and normal kidney tissue. The KEGG pathway enrichment analysis was completed by DEGs, and the figures were generated with the packages “ggplot2” and “enrich plot”.

### 2.3 WGCNA network construction and data analysis

Gene co-expression networks of DN patients were constructed based on the GSE96804 microarray dataset by the “WGCNA” package (16). The soft-thresholding power was five when 0.9 was used as the correlation coefficient threshold, and 50 was chosen as the minimum number of genes in modules. To merge possible similar modules, we defined 0.25 as the threshold for cutting height. A heatmap between the correlation between modules and DN was drawn, and the ME-brown gene module was the most related to DN.

### 2.4 Secreted genes download

729 secreted genes are available for the HPA database (https://www.proteinatlas.org). Venn diagram (https://bioinfogp.cnb.csic.es/tools/venny/index.html) demonstrates the genes which are commonly associated with the 3 datasets (DEGs, WCANA, and secreted to blood genes). In the common genes, we further filtered the core secretory genes by using different machine algorithms (Lasso and SVM-RFE algorithm).

### 2.5 Lasso algorithm and SVM-RFE algorithm data analysis

Lasso logistic regression is a machine learning process that determines covariates by seeking the λ value that minimizes the classification error (17). The “glmnet” package was utilized to structure the LASSO model. Meanwhile, With SVM-RFE, an approach for building machine training on support vector machines, we detect the optimal variables by decimating the feature vectors created by svm (18). Recursive features of differential genes were acquired and erased by running the “e1071 package”, and the research was conducted by applying the Lapply function to sort all the features of the training set. Ultimately, the error rate is minimized and the hub gene is eventually obtained.

### 2.6 Presentation of hub genes

The common genes derived from these two machine algorithms are demonstrated by the Venn diagram, heat maps, line plots, and deviation plots.

### 2.7 Biomarker expression validation and clinical relevance

As illustrated in our previous research, the expression of biomarkers was confirmed by using the Nephroseq database (https://www.nephroseq.org/resource/main.html) (19). Meanwhile, by using the database, biomarker expression and renal function data were analyzed for correlation.

### 2.8 Animal experiments

The STZ-induced DN mouse model was elucidated in detail in our previous research (19), and among them, there were 5 mice in the control group (Ctrl) and 5 mice in the diabetic nephropathy group (DN). Following the successful construction of the DN mouse model, we conduct the collection of experimental animals. The research was approved by the Ethics Committee of Qilu Hospital, Shandong University (Approval No: KYLL-2020(KS)-030).

### 2.9 Western blot

The experimental operation of Western Blot was as described (20). The main antibodies are described as follows: APOC1(1:2000, Abcam, USA), GAPDH (1:4000; Proteintech Group, China).

### 2.10 IHC and IF

Immunohistochemistry and immunofluorescence of kidney tissue sections as previously described (21) The main antibodies are described as follows: APOC1(1:200, Abcam, USA), Goat anti-Rabbit IgG Dy-Light 488 (1:500; Abbkine Scientific Company, USA).

### 2.11 ELISA experiment

We have collected serum specimens from DN patients and healthy. Detection of biomarkers in serum with commercial Elisa kits, ELISA method, in DN patients and healthy. Follow the experimental steps in the Elisa kit instructions to detect the expression level of the marker in the serum (Apolipoprotein CI ELISA kit, Abcam, ab108808, USA).

### 2.12 ROC

The “PROC” package was used to construct Receiver Operating Characteristic (ROC) curves to characterise hub gene to evaluate the diagnostic value of DN, as previously described (19).

### 2.13 Statistical analyses

Data are expressed as mean ± SEM. Software R4.1.2 was used to draw the research Figures. GraphPad Prism 6.01 software was used in statistical data analysis. Between the two groups, Student’s t-test was used if the data matched the normal distribution, and the Kruskal-Wallis test was used for non-normally distributed data. For statistical analysis of the correlation between the two characters, the Spearman test was applied. Statistical significance was set at P < 0.05, *P < 0.05, **P < 0.01, ***P < 0.001.

## 3 Results

### 3.1 Characterisation of genes for DN using GSE96804 microarray data

**Figure 1:**
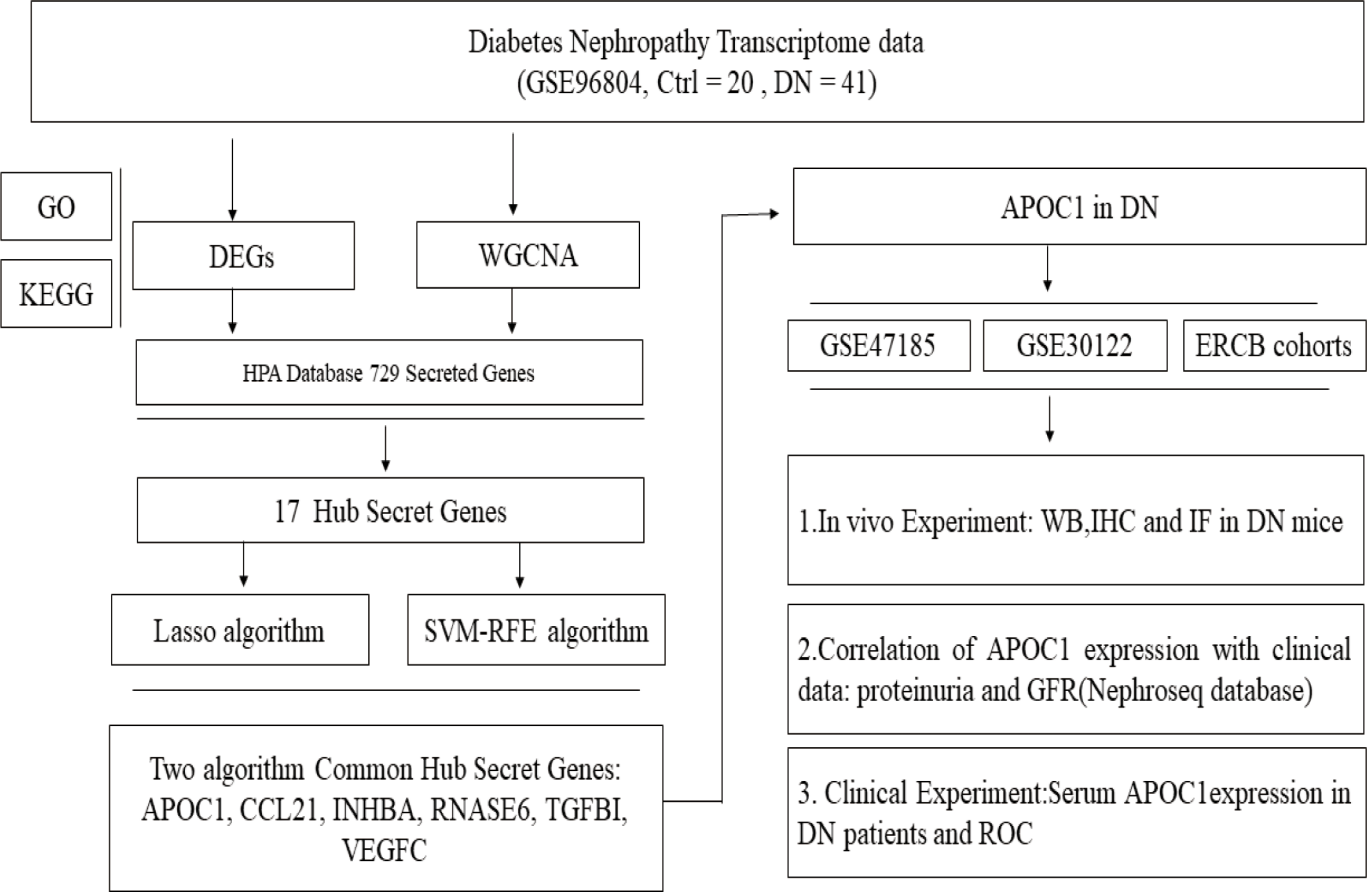
Research flow chart.

**Figure 2:**
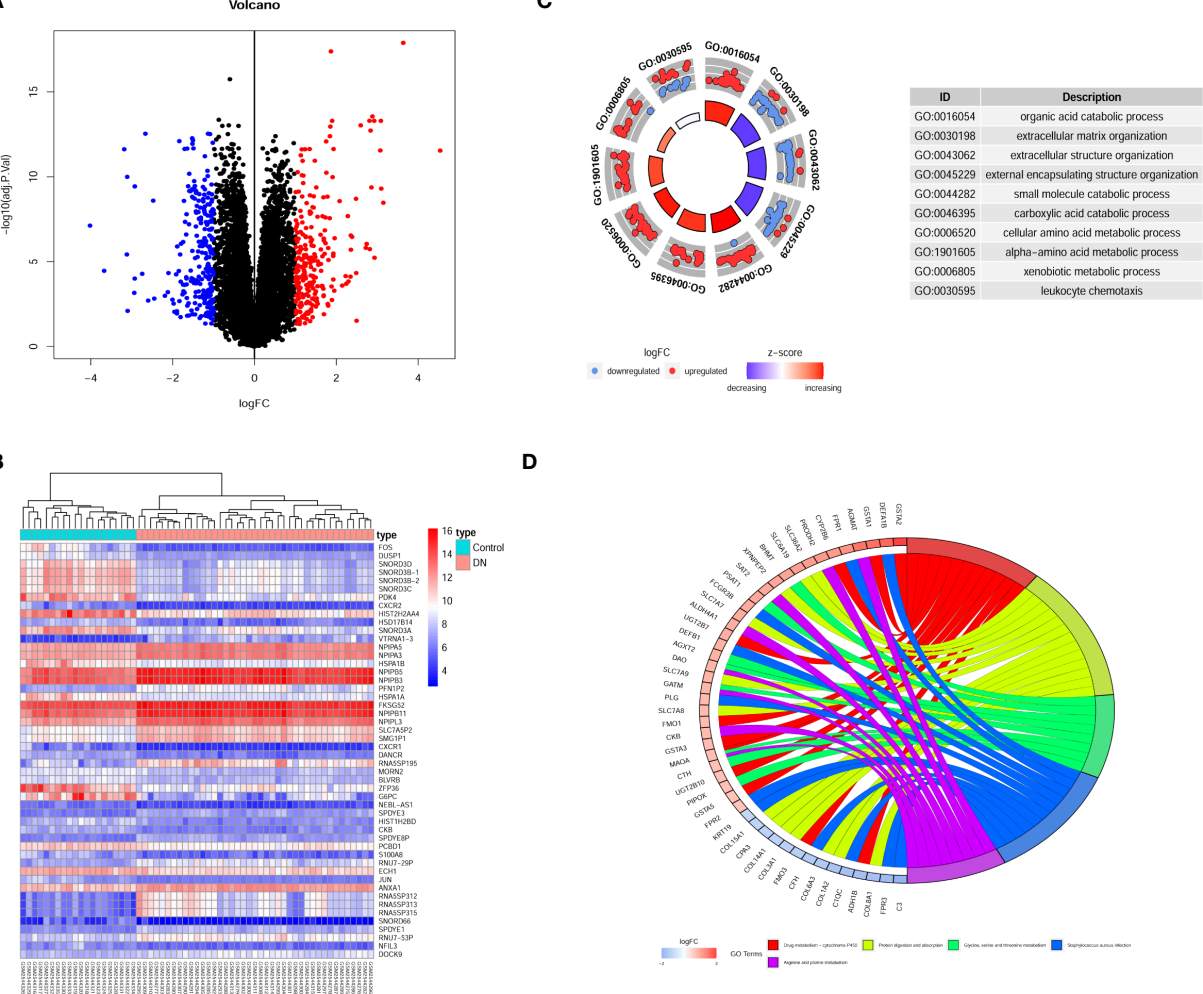
Gene recognition and function enrichment of DEGs in GSE96804 database. **(A)** Volcano-map of DEGs (DEGs: |log2FC| > 1, adjusted P<0.05). **(B)** Heatmap of the DEGs. **(C)** Circle map of GO enrichment analysis. **(D)** Circos map of the KEGG enrichment analysis. DEGs, differentially expressed genes; GO, Gene Ontology; KEGG, Kyoto Encyclopedia of Genes and Genomes.

The experimental design was illustrated in **Figure 1**. Compared to transcripts of controls, 504 DEGs were identified by patients, respectively. Our analysis of the results is summarized in the volcano plots, which reflect that 257 genes are up-regulated in DN and 247 genes are down-regulated in DN (**Figure 2A**). In the illustration, red represents up-regulated and green indicates down-regulated genes. Results demonstrated two clusterings of this data, namely the clusters Control and DN which represents the control group and the DN patients in the heatmap (**Figure 2B**). Analysis of GO in DEGs determined shared GO terms linked to organic acid catabolic processes, and extracellular matrix organization (**Figure 2C**). Enrichment pathways to KEGG are associated with the following: Arginine and proline metabolism, Glycine, serine and threonine metabolism, and Protein digestion and absorption (**Figure 2D**).

### 3.2 Hub gene screening for DN by WGCNA

**Figure 3:**
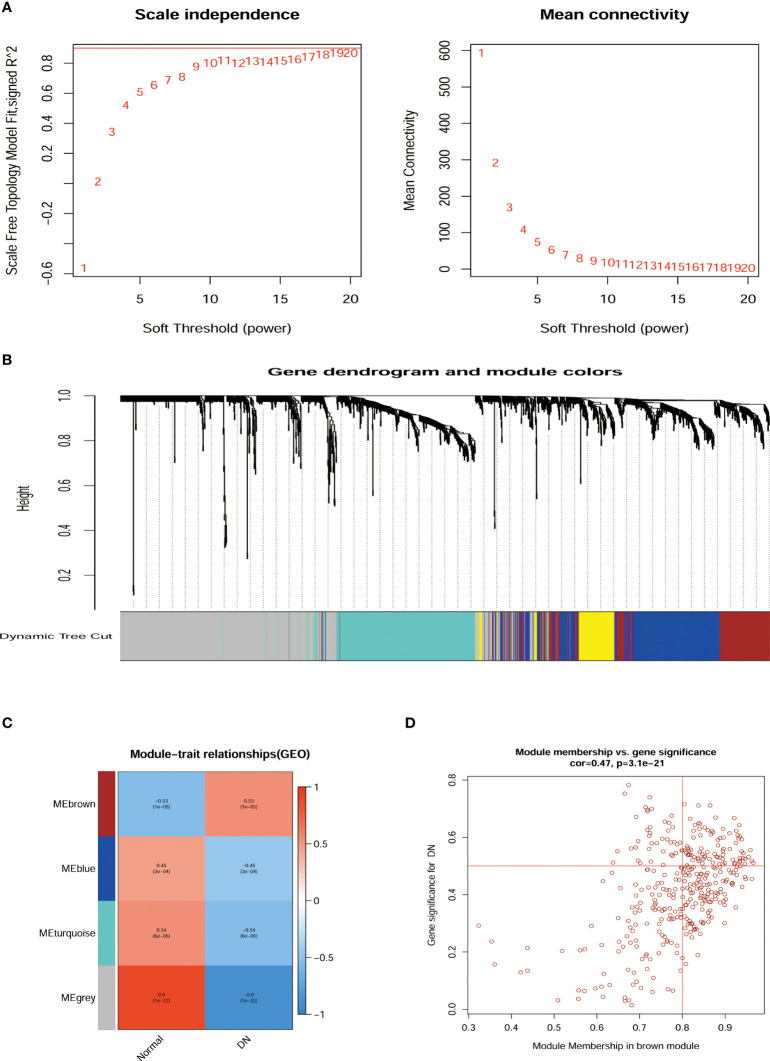
Relationship of hub gene modules and DN phenotypes by WGCNA. **(A)** Network topology analysis at different soft threshold powers and network connectivity validation at different weighting factors. **(B)** Cluster Dendrogram of modules colors were constructed with all the differentially expressed genes. **(C)** MEs correlated with diagnosis for DN. **(D)** Scatterplots of gene significance for DN Module membership in brown module. ME, Module eigengenes; WGCNA, weighted gene co-expression network analysis.

The network topologies for the analysis of various soft threshold powers were identified and the choice of 11 to structure the joint expression network was considered reasonable (**Figure 3A**). The similarity in gene expression is ascertained by pair-weighting correlation metrics, and clustering is performed using topological overlapping metrics. Gene modules are marked with color at the bottom (**Figure 3B**). Pearson correlation coefficients for ME and disease were calculated for all modules demonstrating the intimate characteristics of the modules with DN. ME-brown (R = 0.53, P = 1e-05) potentially represented particular features of DN patients (**Figure 3C**). Furthermore, we observed that the correlation coefficient between the GS of DN and the module members was high in brown modules (R = 0.47, = 3.1e-21, **Figure 3D**). There was potential biological relevance to heightened co-expression of the genes in the ME-brown module.

### 3.3 Screening of hub secretory genes for DN by machine algorithms

**Figure 4:**
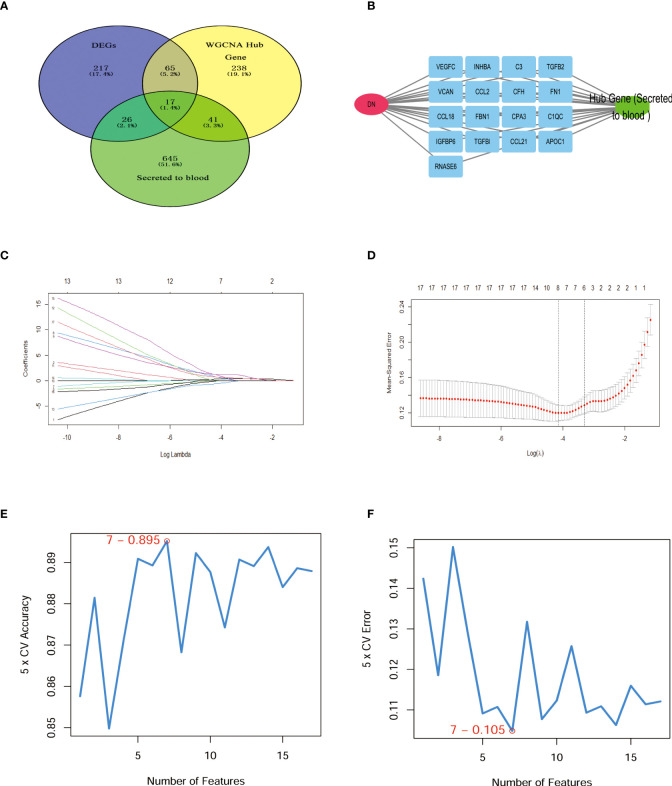
Hub secret genes selection in DN. **(A)** Venn diagram demonstrating the hub genes for the different algorithms. (DEGs, WCANA, and secreted to blood genes). **(B)** 17 characteristically secret genes in DN patients. **(C, D)**Biomarker secret genes were selected by Lasso algorithm from the 17 potential hub genes. **(E, F)** Biomarker secret genes were detected for DN by SVM-RFE algorithm from the 17 potential hub genes (the accuracy and the error rate of the SVM model). Lasso, Least absolute shrinkage and selection operator; SVM-RFE, support vector machine recursive feature elimination.

Venn diagram illustrating common genes across algorithms, filtering for 17 potential secretory genes that may be functionally essential in DN (**Figure 4A**). By using 2 machine algorithms, Lasso and SVM-RFE, to recognize the characteristic genes of DN. The 17 secreted genes are displayed in **Figure 4B**. By using 2 machine algorithms, LASSO and SVM-RFE, characteristic genes of the DN were identified again. The Lasso algorithm filtered out 9 potential hub genes (**Figures 4C, D**), while the SVM algorithm filtered out 7 potential hub genes (**Figures 4E, F**).

### 3.4 Expression of 6 secretory genes in DN

**Figure 5:**
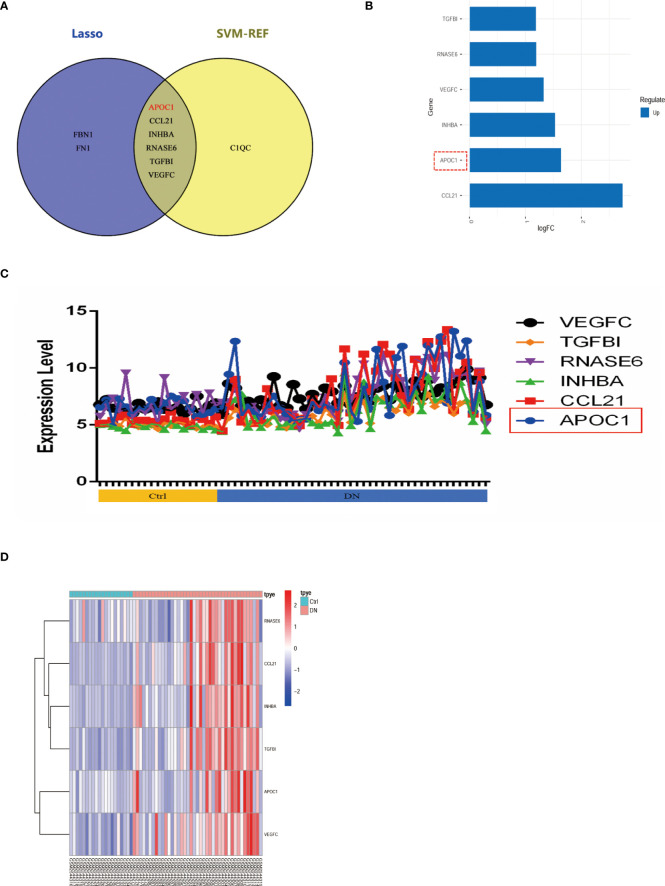
6 potential secretory genes were obtained in GSE96804 by machine algorithms. **(A)** The intersection of genes obtained by the two machine algorithms (SVM-RFE and Lasso algorithms). **(B)** Deviation plots showed the expression of six secreted genes in DN. **(C)** Folding line graph illustrates the different expression of 6 hub genes. **(D)** Heat plots revealed elevated expression of six secreted genes in DN.

The Venn diagram illustrates 2 machine algorithms obtained common 6 hub secretory genes (APOC1, CCL21, INHBA, RNASE6, TGFBI, VEGFC, **Figure 5A**). Furthermore, the expression of the six genes in the GSE96804 cohort is illustrated by heatmap, line graphs, and deviation plots (**Figures 5B–D**). The results revealed that 6 secretory gene generators screened for the research were significantly more over-expressed in the diabetic nephropathy population.

### 3.5 Associated expression of APOC1 in DN

**Figure 6:**
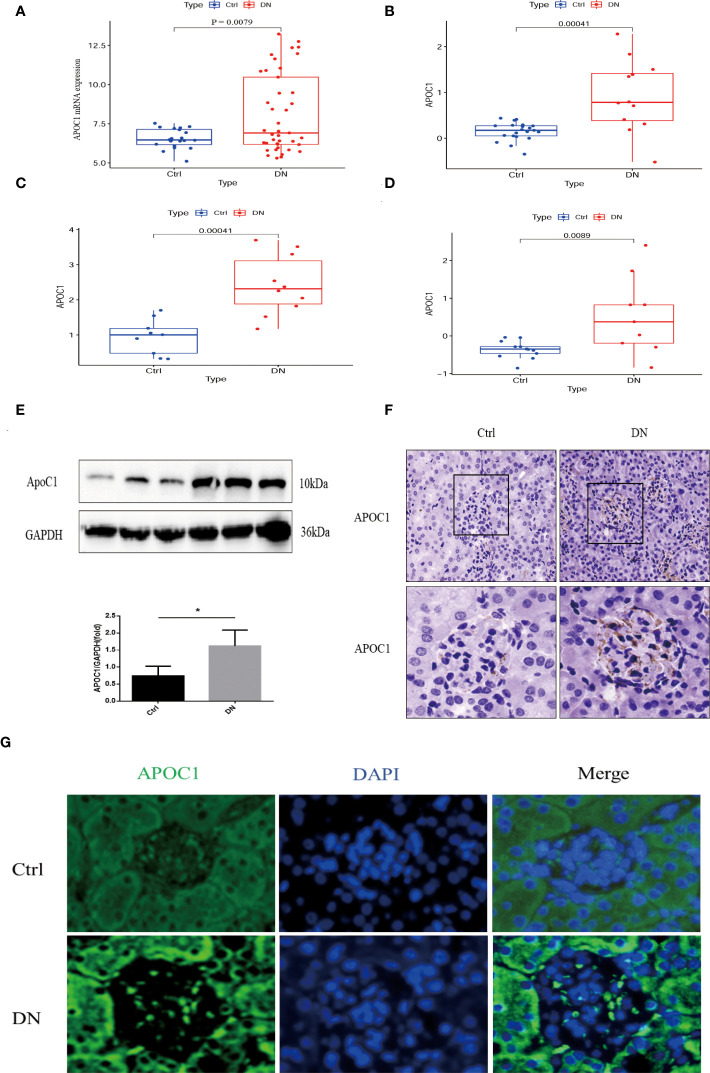
Exhibition of the expression of APOC1 in DN. **(A–D)** APOC1 manifested significantly higher expression in different cohorts of DN patients. [**(A)**: GSE96804, Ctrl=20, DN=41, **(B)** GSE47185, Ctrl=21, DN=12, **(C)** ERCB Nephrotic Syndrome Tublnt cohorts in Nephroseq database, Ctrl=9, DN=10, GSE 30122, Ctrl=13, DN=9]. **(E)** Representative Western Blot indicates APOC1 expression to be higher in different mice, Ctrl (n = 4) or DN (n = 4). **(F)** IHC reveals increased expression of APOC1 on glomeruli of mice with DN (Bar = 20 μm). **(G)** Representative protein immunofluorescence of APOC1 in the glomeruli of DN (Bar = 20 μm.). (Data presented as mean ± SEM, *P < 0.05).

APOC1 expression is elevated in patients with diabetic nephropathy through multiple cohorts of the experimental GEO database (GSE96804, GSE47185, GSE30122, and the ERCB Nephrotic Syndrome Tublnt cohorts in Nephroseq database, **Figures 6A–D**). ApoC1 expression was elevated in the kidney tissue of mice with DN by Western blot (P < 0.05, **Figure 6E**). What’s more, we revealed that APOC1 was expressed predominantly in the glomerulus by immunohistochemistry of mouse kidney tissue (**Figure 6F**). These measurements were confirmed by tissue immunofluorescence (**Figure 6G**).

### 3.6 Correlation of APOC1 expression with clinical databases

**Figure 7:**
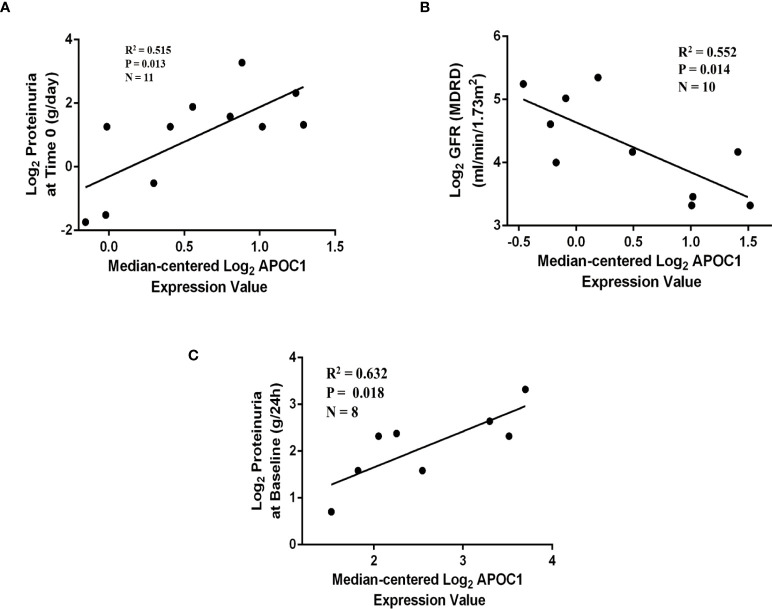
Correlation between APOC1 expression and proteinuria and GFR in Nephroseq database. **(A)** Correlation between APOC1 expression and proteinuria in Schmid diabetes tubint cohorts (R^2^ = 0.515, P = 0.013). **(B)** Correlation between APOC1 expression and GFE in Woroniecka Diabetes Tublnt cohorts (R^2^ = 0.552, P = 0.014). **(C)** Correlation between APOC1 expression and GFE in ERCB Nephrotic Syndrome Tublnt cohorts (R^2^ = 0.632, P = 0.018).

Correlations between APOC1 and the clinical information were validated by employing multiple cohorts from the Nephroseq database. Outcomes demonstrated the APOC1 expression was positively correlated with proteinuria in Schmid diabetes tubint cohorts (R^2^ = 0.515, P = 0.013, **Figure 7A**). However, associations of APOC1 expression are negatively correlated with GFR in Woroniecka Diabetes Tublnt cohorts (R^2^ = 0.552, P = 0.014, **Figure 7B**). Additionally, in ERCB Nephrotic Syndrome Tublnt cohorts, APOC1 expression was positively correlated with proteinuria (R^2^ = 0.632, P = 0.018, **Figure 7C**).

### 3.7 Plasma expression of APOC1 in DN patients and ROC curve analysis

**Figure 8:**
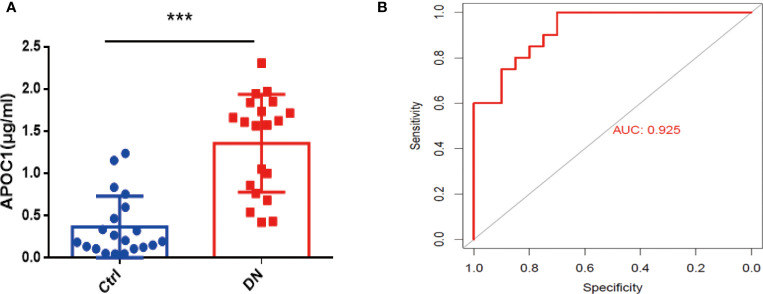
Serum APOC1 expression in DN patients and ROC. **(A)** APOC1 expression in serum (Ctrl n = 20, DN n = 20). **(B)** ROC curve of serum APOC1 expression in DN (AUC = 92.5%, sensitivity = 95%, and specificity = 97%, P < 0.001). Ctrl, Healthy population; DN, Diabetic nephropathy patients. ***P < 0.001.

Altogether 20 healthy and 20 DN patients were enrolled in the research, and the Baseline details were presented in **Table 1**. Significantly, Elisa results demonstrated that APOC1 expression in the serum of DN patients was 1.358±0.1292μg/ml, compared to 0.3683±0.08119μg/ml in the healthy population (**Figure 8A**). APOC1 was significantly elevated in the sera of DN patients and the difference was statistical significant (P > 0.001). Furthermore, APOC1 diagnostic effectiveness for DN as demonstrated by ROC curves (AUC = 92.5%, sensitivity = 95%, and specificity = 97%, P < 0.001, **Figure 8B**).

**Table 1:** Baseline characteristics.

Characteristic	Ctrl (n = 20)	DN (n = 20)
Age (year)	46.10 ± 2.625	49.30 ± 3.361
Sex (Female/male)	11/9	7/13
SBP (mmHg)	122.9 ± 1.832	131.8 ± 5.333
DBP (mmHg)	70.70 ± 1.223	75.95 ± 2.300
eGFR (ml/min1.73m^2^)	103.0 ± 5.345	106.4 ± 9.705
Cr (μmoI/L)	72.40 ± 3.438	82.80 ± 9.288
ACR	0.0035 ± 0.001313	0.2425 ± 0.1029^*^
CHO (mmol/l)	5.111 ± 0.2631	4.603 ± 0.3581
TG (mmol/l)	1.459 ± 0.2988	2.018 ± 0.3723
UA (mmol/l)	306.9 ± 13.10	329.9 ± 20.37
APOC1(μg/ml)	0.3683 ± 0.08119	1.358 ± 0.1292^***^

SBP, Systolic Blood Pressure; Diastolic Blood Pressure; eGFR, Estimated Glomerular Filtration Rate; Cr, Creatinine; ACR, Albumin/Urine Creatinine Ratio; CHO, Cholesterol; TG, Triglyceride; UA, Uric Acid; Ctrl, Healthy population (n = 20); DN, Diabetic nephropathy patients (n = 20). Ctrl vs DN; *P < 0.05; ***P < 0.001.

## 4 Discussion

DN is considered to the most serious complication of diabetes and imposes a substantial financial burden on individuals and society (22). It is vital to diagnose DN early to improve the prognosis of patients with DN and reduce the financial burden (23). However, the most dominant clinical indicators for the diagnosis of DN are still UACR and eGFR, in clinical practice (24). Previous studies have demonstrated that damage to the kidney, such as endothelial damage, tubulointerstitial dilatation, and interstitial fibrosis, has already occurred before the appearance of albuminuria in patients with DN (25). The abnormalities in molecular markers usually precede the clinical symptoms of the disease (26, 27). Therefore, the urgent challenge is to identify suitable, stable, and easily detectable biomarkers for DN diagnosis.

Microarrays have been extensively implemented in medical research, such as biomarkers for disease diagnosis, and prognosis (28, 29). Consequently, we investigated the differential genes in the kidney tissue of diabetic nephropathy and healthy people by microarray transcriptome analysis (**Figure 2**). The research demonstrated that 257 up-regulated and 247 down-regulated genes were compared to normal kidney tissue. Furthermore, we also screened for gene modules closely correlated with diabetic nephropathy by the WGCNA method (**Figure 3**). 17 secretory genes were obtained in the differential and Me-Brown modules (**Figure 3**, **4**), which may have an essential role in DN.

Our investigation further screened for core secretory genes in diabetic nephropathy using the Lasso and SVM-RFE machine learning algorithms, which identified a total of six potential core genes (**Figures 4**, **5**). Among the six secreted genes, APOC1 is newly identified as a member of the lipoprotein family and is closely associated with lipid metabolism and immune inflammation. Our research demonstrated elevated expression of APOC1 in DN. Additionally, APOC1 expression was also confirmed by other transcriptome microarray data (**Figure 6**). Lipid metabolism disorders and immunoinflammatory responses are critical in the development and progression of DN patients (30, 31), which means that APOC1 may be also involved in the development of DN.

APOC1 has been implicated in the progress of many diseases such as malignancy (32), atherosclerosis (33), and Alzheimer’s disease (34). More importantly, APOC1 is closely associated with cell proliferation, apoptosis, and immune inflammation (35). Recent research also has identified ApoC1 which promotes renal clear cell carcinoma metastasis through activation of the STAT3 pathway (36) and is a potential novel diagnostic and prognostic marker for clear cell renal carcinoma (37). Animal experiments are employed to confirm the results of research. In vivo, we also demonstrated that APOC1 expression was significantly increased in diabetic nephropathy kidney tissues, mainly in the glomerulus, using a mouse model of diabetic nephropathy (**Figure 6**). Currently, our team are also conducting functional and mechanistic research on the role of APOC1 in DN.

Interestingly, we also conducted a correlation analysis between APOC1 and clinical data. we investigated the correlation of APOC1 expression with urinary protein and eGFR in DN patients through the Nephroseq database (**Figure 7**). For further evidence, we collected blood samples from 20 patients with DN and 20 healthy. We assayed the expression level of APOC1 in serum by Elisa assay. The outcome showed that APOC1 expression was significantly higher in DN patients and had an excellent diagnostic efficacy for DN (**Figure 8**). Therefore, we concluded that APOC1 may be a novel biomarker for DN. Nevertheless, many deficiencies remain for our research. The role and mechanism of APOC1 in the development of DN is still unclear. The diagnostic efficacy of APOC1 for DN still needs to be demonstrated in multicentre research. Additionally, APOC1 expression and the prognosis of DN patients still need more prospective investigation.

In conclusion, elevated glomerular and serum expression of APOC1 in DN was identified for the first time through bioinformatics, machine learning, animal model experiments, and clinical data. APOC1 was demonstrated to be a novel and potential biological diagnostic marker for DN, but additional prospective research remains needed to demonstrate its diagnostic value.

## Data availability statement

The datasets presented in this study can be found in online repositories. The names of the repository/repositories and accession number(s) can be found in the article/**Supplementary Material**.

## Ethics statement

The studies involving human participants were reviewed and approved by the Ethics Committee of Qilu Hospital, Shandong University (Approval No: KYLL-2020(KS)-030). The patients/participants provided their written informed consent to participate in this study. The animal study was reviewed and approved by the Ethics Committee of Qilu Hospital, Shandong University (Approval No: KYLL-2020(KS)-030).

## Author contributions

KY: Original draft and Writing, SL, and CW: Drawing diagrams, LL, XF, L F: Animal experiments and Clinical Data Collection, YZ: Clinical Data Collection, HL, HY, JS: Methodology, XY: Review and editing. All authors contributed to the article and approved the submitted version.

## References

[1] Ghaderian SB Hayati F Shayanpour S Beladi Mousavi SS. Diabetes and end-stage renal disease; a review article on new concepts. J Renal Inj Prev (2015) 4:28–33. doi: 10.12861/jrip.2015.07 26060834 PMC4459725

[2] Cheng HT Xu X Lim PS Hung KY. Worldwide epidemiology of diabetes-related end-stage renal disease, 2000-2015. Diabetes Care (2021) 44:89–97. doi: 10.2337/dc20-1913 33203706

[3] Schrauben SJ Shou H Zhang X Anderson AH Bonventre JV. Association of multiple plasma biomarker concentrations with progression of prevalent diabetic kidney disease: Findings from the chronic renal insufficiency cohort (CRIC) study. J Am Soc Nephrol. (2021) 32:115–26. doi: 10.1681/ASN.2020040487 PMC7894671 33122288

[4] Vasquez-Rios G Coca SG. Predictors of kidney disease progression in diabetes and precision medicine: Something old, something new, and something borrowed. J Am Soc Nephrol. (2021) 32:2108–11. doi: 10.1681/ASN.2021070945 PMC8729853 34465605

[5] Zhang WR Craven TE Malhotra R Cheung AK Chonchol M Drawz P. Kidney damage biomarkers and incident chronic kidney disease during blood pressure reduction: A case-control study. Ann Intern Med (2018) 169:610–8. doi: 10.7326/M18-1037 PMC6953744 30357395

[6] Barrett T Wilhite SE Ledoux P Evangelista C Kim IF Tomashevsky M. NCBI GEO: archive for functional genomics data sets–update. Nucleic Acids Res (2012) 41:D991–5. doi: 10.1093/nar/gks1193 PMC3531084 23193258

[7] Huang M Zhu Z Nong C Liang Z Ma J Li G. Bioinformatics analysis identifies diagnostic biomarkers and their correlation with immune infiltration in diabetic nephropathy. Ann Transl Med (2022) 10:669. doi: 10.21037/atm-22-1682 35845512 PMC9279778

[8] Zhao Z He S Yu X Lai X Tang S Mariya MEA. Analysis and experimental validation of rheumatoid arthritis innate immunity gene CYFIP2 and pan-cancer. Front Immunol (2022) 13:954848. doi: 10.3389/fimmu.2022.954848 35898498 PMC9311328

[9] Li D Yu K Feng F Zhang Y Bai F Zhang Y. Hydroxychloroquine alleviates renal interstitial fibrosis by inhibiting the PI3K/Akt signaling pathway. Biochem Biophys Res Commun (2022) 610:154–61. doi: 10.1016/j.bbrc.2022.04.058 35462097

[10] Huang W Wang BO Hou YF Fu Y Cui SJ Zhu JH. JAML promotes acute kidney injury mainly through a macrophage-dependent mechanism. JCI Insight (2022) 7:1–19. doi: 10.1172/jci.insight.158571 PMC9431718 35708906

[11] Bullen AL Katz R Jotwani V Garimella PS Lee AK Estrella MM. Biomarkers of kidney tubule health, CKD progression, and acute kidney injury in SPRINT (Systolic blood pressure intervention trial) participants. Am J Kidney Dis (2021) 78:361–368 e361. doi: 10.1053/j.ajkd.2021.01.021 33857535 PMC8384678

[12] Pan Y Jiang S Hou Q Qiu D Shi J Wang L. Dissection of glomerular transcriptional profile in patients with diabetic nephropathy: SRGAP2a protects podocyte structure and function. Diabetes (2018) 67:717–30. doi: 10.2337/db17-0755 29242313

[13] Ritchie ME Phipson B Wu D Hu Y Law CW Shi W. Limma powers differential expression analyses for RNA-sequencing and microarray studies. Nucleic Acids Res (2015) 43:e47. doi: 10.1093/nar/gkv007 25605792 PMC4402510

[14] Ito K Murphy D. Application of ggplot2 to pharmacometric graphics. CPT Pharmacometrics Syst Pharmacol (2013) 2:e79. doi: 10.1038/psp.2013.56 24132163 PMC3817376

[15] Yu G Wang LG Han Y He QY. clusterProfiler: An r package for comparing biological themes among gene clusters. OMICS (2012) 16:284–7. doi: 10.1089/omi.2011.0118 PMC3339379 22455463

[16] Langfelder P Horvath S. WGCNA: An r package for weighted correlation network analysis. BMC Bioinf (2008) 9:559. doi: 10.1186/1471-2105-9-559 PMC2631488 19114008

[17] Antonacci Y Toppi J Mattia D Pietrabissa A Astolfi L. Single-trial connectivity estimation through the least absolute shrinkage and selection operator. Annu Int Conf IEEE Eng Med Biol Soc (2019) 2019:6422–5. doi: 10.1109/EMBC.2019.8857909 31947312

[18] Zhu YX Huang JQ Ming YY Zhuang Z Xia H. Screening of key biomarkers of tendinopathy based on bioinformatics and machine learning algorithms. PloS One (2021) 16:e0259475. doi: 10.1371/journal.pone.0259475 34714891 PMC8555777

[19] Yu K Li D Xu F Guo H Feng F Ding Y. IDO1 as a new immune biomarker for diabetic nephropathy and its correlation with immune cell infiltration. Int Immunopharmacol. (2021) 94:107446. doi: 10.1016/j.intimp.2021.107446 33581581

[20] Yang H Xie T Li D Du X Wang T Li C. Tim-3 aggravates podocyte injury in diabetic nephropathy by promoting macrophage activation *via* the NF-kappaB/TNF-alpha pathway. Mol Metab (2019) 23:24–36. doi: 10.1016/j.molmet.2019.02.007 30862474 PMC6479760

[21] Liu L Bai F Song H Xiao R Wang Y Yang H. Upregulation of TIPE1 in tubular epithelial cell aggravates diabetic nephropathy by disrupting PHB2 mediated mitophagy. Redox Biol (2022) 50:102260. doi: 10.1016/j.redox.2022.102260 35152003 PMC8844679

[22] Umanath K Lewis JB. Update on diabetic nephropathy: Core curriculum 2018. Am J Kidney Dis (2018) 71:884–95. doi: 10.1053/j.ajkd.2017.10.026 29398179

[23] Barrera-Chimal J Jaisser F. Pathophysiologic mechanisms in diabetic kidney disease: A focus on current and future therapeutic targets. Diabetes Obes Metab (2020) 22 Suppl 1:16–31. doi: 10.1111/dom.13969 32267077

[24] Gross JL de Azevedo MJ Silveiro SP Canani LH Caramori ML Zelmanovitz T. Diabetic nephropathy: Diagnosis, prevention, and treatment. Diabetes Care (2005) 28:164–76. doi: 10.2337/diacare.28.1.164 15616252

[25] Slyne J Slattery C McMorrow T Ryan MP. New developments concerning the proximal tubule in diabetic nephropathy: In vitro models and mechanisms. Nephrol Dial Transplant (2015) 30 Suppl 4:iv60–67. doi: 10.1093/ndt/gfv264 26209740

[26] Flyvbjerg A. The role of the complement system in diabetic nephropathy. Nat Rev Nephrol. (2017) 13:311–8. doi: 10.1038/nrneph.2017.31 28262777

[27] Butler AE Al-Qaissi A Sathyapalan T Atkin SL. Angiopoietin-1: An early biomarker of diabetic nephropathy? J Transl Med (2021) 19:427. doi: 10.1186/s12967-021-03105-9 34645474 PMC8513175

[28] Yanaoka H Nagafuchi Y Hanata N Takeshima Y Ota M Suwa Y. Identifying the most influential gene expression profile in distinguishing ANCA-associated vasculitis from healthy controls. J Autoimmun (2021) 119:102617. doi: 10.1016/j.jaut.2021.102617 33677398

[29] Xu Y Tan Y Zhang X Cheng M Hu J Liu J. Comprehensive identification of immuno-related transcriptional signature for active pulmonary tuberculosis by integrated analysis of array and single cell RNA-seq. J Infect (2022) 2022:534–544. doi: 10.1016/j.jinf.2022.08.017 36007657

[30] Lu J Chen PP Zhang JX Li XQ Wang GH Yuan BY. GPR43 activation-mediated lipotoxicity contributes to podocyte injury in diabetic nephropathy by modulating the ERK/EGR1 pathway. Int J Biol Sci (2022) 18:96–111. doi: 10.7150/ijbs.64665 34975320 PMC8692141

[31] Wu L Liu C Chang DY Zhan R Zhao M Man Lam S. The attenuation of diabetic nephropathy by annexin A1 *via* regulation of lipid metabolism through the AMPK/PPARalpha/CPT1b pathway. Diabetes (2021) 70:2192–203. doi: 10.2337/db21-0050 34103347

[32] Zheng XJ Chen WL Yi J Li W Liu JY Fu WQ. Apolipoprotein C1 promotes glioblastoma tumorigenesis by reducing KEAP1/NRF2 and CBS-regulated ferroptosis. Acta Pharmacol Sin (2022) 2022:2977–2992. doi: 10.1038/s41401-022-00969-5 PMC9622891 35581292

[33] Westerterp M Berbee JF Pires NM van Mierlo GJ Kleemann R Romijn JA. Apolipoprotein c-I is crucially involved in lipopolysaccharide-induced atherosclerosis development in apolipoprotein e-knockout mice. Circulation (2007) 116:2173–81. doi: 10.1161/CIRCULATIONAHA.107.693382 17967778

[34] Pathak GA Zhou Z Silzer TK Barber RC Phillips NR. Two-stage Bayesian GWAS of 9576 individuals identifies SNP regions that are targeted by miRNAs inversely expressed in alzheimer's and cancer. Alzheimers Dement. (2020) 16:162–77. doi: 10.1002/alz.12003 PMC13221707 31914222

[35] Fuior EV Gafencu AV. Apolipoprotein C1: Its pleiotropic effects in lipid metabolism and beyond. Int J Mol Sci (2019) 20:1–25. doi: 10.3390/ijms20235939 PMC6928722 31779116

[36] Li YL Wu LW Zeng LH Zhang ZY Wang W Zhang C. ApoC1 promotes the metastasis of clear cell renal cell carcinoma *via* activation of STAT3. Oncogene (2020) 39:6203–17. doi: 10.1038/s41388-020-01428-3 32826950

[37] Cui Y Miao C Hou C Wang Z Liu B. Apolipoprotein C1 (APOC1): A novel diagnostic and prognostic biomarker for clear cell renal cell carcinoma. Front Oncol (2020) 10:1436. doi: 10.3389/fonc.2020.01436 32974161 PMC7468425

